# Peritoneal VEGF-A expression is regulated by TGF-β1 through an ID1 pathway in women with endometriosis

**DOI:** 10.1038/srep16859

**Published:** 2015-11-18

**Authors:** Vicky J. Young, Syed F. Ahmad, Jeremy K. Brown, W. Colin Duncan, Andrew W. Horne

**Affiliations:** 1 MRC Centre for Reproductive Health, The University of Edinburgh, Queen’s Medical Research Institute, Edinburgh EH16 4TJ, UK

## Abstract

VEGF-A, an angiogenic factor, is increased in the peritoneal fluid of women with endometriosis. The cytokine TGF-β1 is thought to play a role in the establishment of endometriosis lesions. Inhibitor of DNA binding (ID) proteins are transcriptional targets of TGF-β1 and ID1 has been implicated in VEGF-A regulation during tumor angiogenesis. Herein, we determined whether peritoneal expression of VEGF-A is regulated by TGF-β1 through the ID1 pathway in women with endometriosis. VEGF-A was measured in peritoneal fluid by ELISA (n = 16). VEGF-A and ID1 expression was examined in peritoneal biopsies (n = 13), and primary peritoneal and immortalized mesothelial cells (MeT5A) by immunohistochemistry, qRT-PCR and ELISA. VEGF-A was increased in peritoneal fluid from women with endometriosis and levels correlated with TGF-β1 concentrations (*P* < 0.05). VEGF-A was immunolocalized to peritoneal mesothelium and TGF-β1 increased *VEGFA* mRNA (*P* < 0.05) and protein (*P* < 0.05) in mesothelial cells. ID1 was increased in peritoneum from women with endometriosis and TGF-β1 increased concentrations of *ID1* mRNA (*P* < 0.05) in mesothelial cells. VEGF-A regulation through ID1 was confirmed by siRNA in MeT5A cells (*P* < 0.05). Our data supports role for ID1 in the pathophysiology of endometriosis, as an effector of TGFβ1 dependent upregulation of VEGF-A, and highlights a novel potential therapeutic target.

Endometriosis is a hormone-dependent benign disorder characterized by the presence of ectopic endometrial tissue commonly found on the pelvic peritoneum^1^,^2^. It is estimated to effect between 2–10% of women of reproductive age and it is associated with chronic pelvic pain and infertility^1^. Endometriosis is currently managed surgically or medically, however lesions reoccur in up to 75% of surgical cases and medical treatments have undesirable side effects^3^. The etiology of endometriosis is unclear. To date, the majority of research has centred on changes within the eutopic and ectopic endometrium of women with endometriosis, but there is now increasing evidence that the peritoneal mesothelial cells may contribute to the development and maintenance of endometriosis lesions^4^.

Angiogenesis is a crucial step in the development of endometriosis lesions. At a macroscopic level, lesions have been shown to be highly vascularized with new vessels developing from the surrounding peritoneum^5^. Vascular endothelial growth factor-A (VEGF-A), a potent angiogenic factor, is known to be increased in the peritoneal fluid of women with endometriosis compared to women without disease^6^,^7^. Levels correlate significantly with the stage of disease and appear to be hormonally regulated^8^. Reported sources of VEGF-A include ectopic endometrium and peritoneal macrophages^9^,^10^.

The largest cell population within the peritoneum, however, is peritoneal mesothelial cells and these cells intimately interact with the ectopic endometrium during the establishment of endometriotic lesions^4^. TGF-β1 is an established regulator of VEGF-A expression in several cell types and this pathway has been implicated in neoangiogenesis of several cancers^11^. Aberrant TGF-β1 signaling plays a critical role in the development of endometriosis lesions, which shares several parallels with tumorigenesis. Several studies have shown that TGF-β1 is increased in the peritoneal fluid, peritoneum and ectopic endometrium of women with endometriosis^12^,^13^,^14^,^15^, suggesting that the same over production of TGF-β1 that is seen in tumours and the surrounding stroma is also true for endometriosis lesions and the surrounding peritoneum. Furthermore, the importance of local TGF-β1 action is highlighted by changes in the expression of TGF-β signaling targets in the peritoneum adjacent to endometriosis lesions^12^,^13^,^14^,^15^.

One TGF-β-signaling target linked to the transcriptional regulation of angiogenesis is inhibitor of DNA binding protein 1 (ID1). ID1 is overexpressed in over 20 types of human cancers^16^ and we have recently shown that it is expressed in the peritoneum of women with endometriosis and regulated by TGF-β1^12^. ID1 has recently been described as an oncogene and much of this evidence is based on ID1 regulation of VEGF-A, with an overexpression of ID1 leading to increases in *VEGFA* gene transcription and hence angiogenesis^17^,^18^. The role of ID1 in angiogenesis is further backed up with evidence that tumours failed to grow and/or metastasise in ID1 +/−; ID3 −/− mice due to poor vascularisation^19^. We hypothesized that the peritoneal mesothelium is a source of VEGF-A in endometriosis and that TGF-β1 regulates the expression of VEGF-A in the peritoneal mesothelial cell through the ID1 pathway, supporting lesion vascularization. Herein, we investigate the expression of VEGF-A in the peritoneal mesothelium and determine if it is regulated by TGF-β1 through an ID1 pathway in women with endometriosis.

## Results

### Increased concentrations of VEGF-A in the peritoneal fluid of women with endometriosis correlate with TGF-β1 concentrations

**Figure 1:**
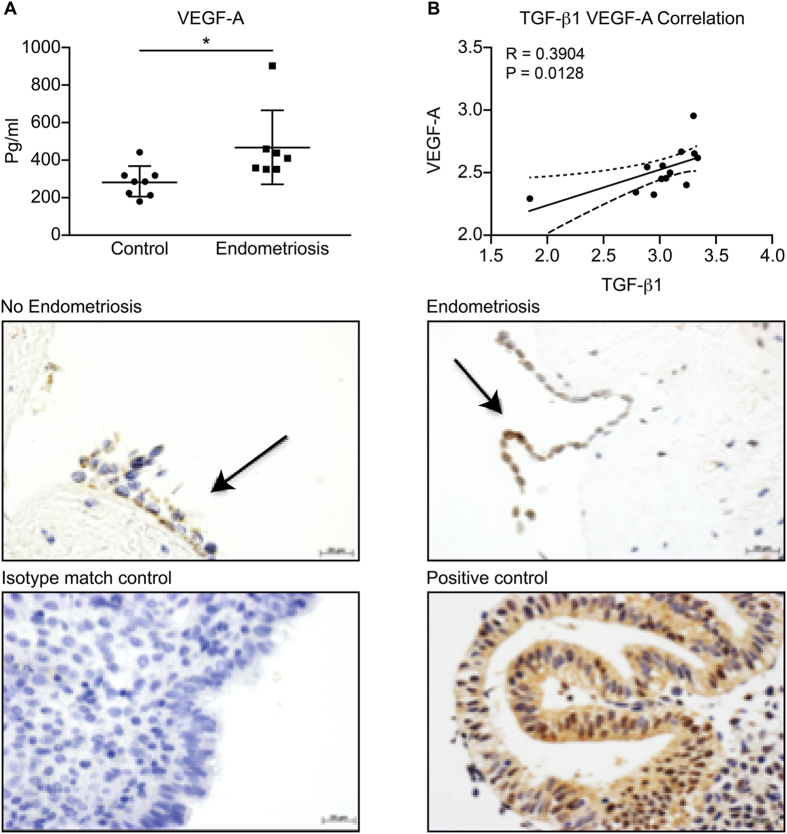
VEGF-A protein concentrations are increased in peritoneal fluid from women with endometriosis compared to women without (**A**) and levels of VEGF-A positively correlate with TGF-β1 concentrations (**B**) (*p < 0.05 unpaired t*-*test, n = 8 each group). Immunohistochemistry of paraffin-embedded sections shows presence and localization of VEGF-A in peritoneal mesothelial cells of women with and without endometriosis, arrows indicate the peritoneal mesothelial cells (**C**). Endometrial tissue was used as positive control and no staining was observed in the isotype match control *(n* = *3 each group)*.

VEGF-A concentrations are increased in the peritoneal fluid of women with endometriosis compared to women without endometriosis (*P* < 0.05; Fig. 1A). We have reported in our previous studies that TGF-β1 concentration was significantly increased in the peritoneal fluid of women with endometriosis compared to women without endometriosis^13^. There was a significant positive correlation between the concentrations of VEGF-A and those of TGF-β1 in the peritoneal fluid from women with and without endometriosis (*R* = 0.39, *P* < 0.05; Fig. 1B). Immunohistochemistry shows VEGF-A to be localised to the peritoneal mesothelial cells of women with and without endometriosis (Fig. 1C).

### TGF-β1 regulates VEGF-A expression in peritoneal mesothelial cells

**Figure 2:**
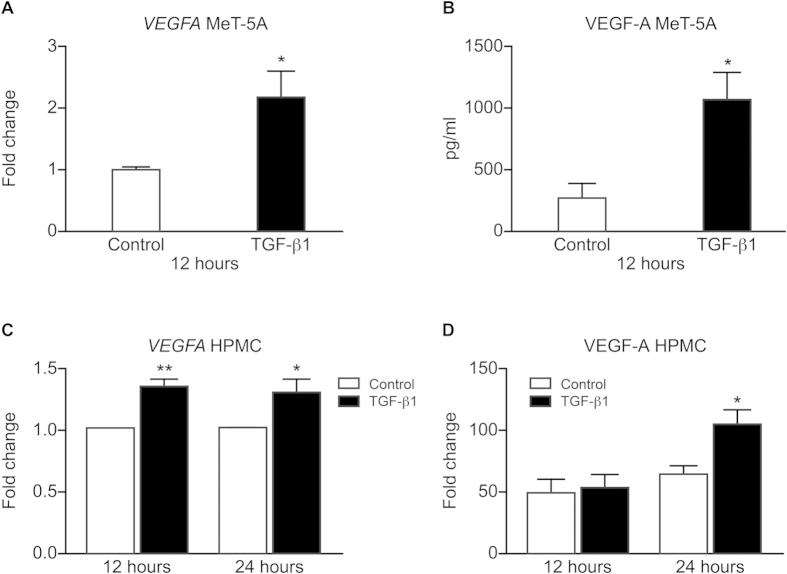
Effect of TGF-β1 on VEGF-A mRNA and protein expression in HPMC and MeT-5A cells. TGF-β1 increased *VEGFA* mRNA (**A**) and VEGF-A protein expression (**B**) in the HPMC obtained from women with endometriosis at 12 hours (*p < *0.05 **paired t-test**, n* = *6)*. TGF-β1 increased *VEGFA* mRNA at 12 and 24 hours (**C**) and significantly increased VEGF-A secretion into the extracellular environment in MeT-5A cells at 24 hours (**D**) (*p < 0.05 **unpaired t-test**, **p < *0.01 **unpaired t-test**, n* = *3 each group)*.

To address the question of whether TGF-β1 regulates VEGF-A expression in peritoneal mesothelial cells, we exposed HPMC and MeT-5A cells to physiological concentrations of TGF-β1 (2 ng/ml). TGF-β1 increased *VEGFA* mRNA expression (*P* < 0.05; Fig. 2A) and extracellular VEGF-A protein concentrations in HPMC at 12 hours (*P* < 0.05; Fig. 2B). In addition we confirmed and extended data from the HPMC by demonstrating TGF-β1 regulates *VEGFA* mRNA expression and VEGF-A protein secretion in the MeT-5A cell line (*P* < 0.05; Fig. 2C,D).

### TGF-β1 target ID1 has a peritoneal localization and its expression is increased in the peritoneum of women with endometriosis

**Figure 3:**
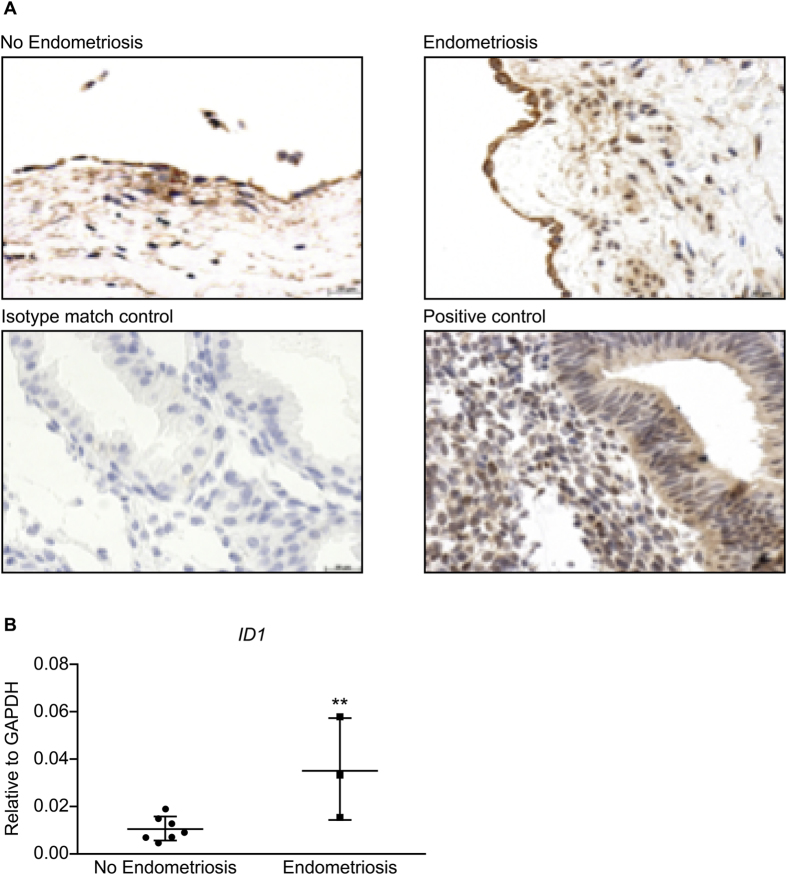
Immunohistochemistry shows presence and localization of ID1 in mesothelial and endothelial cells of the peritoneum of women with and without endometriosis (**A**). Endometrial tissue was used as positive control and no staining was observed in the isotype match control *(n* = *3 each group)*. Peritoneum from women with endometriosis expressed significantly higher levels of *ID1* mRNA when compared to women without endometriosis (**B**) (**p < *0.01 **unpaired t-test**, n* = *8 no endometriosis, n* = *3 endometriosis)*.

The transcriptional regulatory protein ID1 is a known target of TGF-β1 and we found ID1 protein to be localized to the mesothelial, stromal and endothelial cells of the peritoneum (Fig. 3A). To investigate if ID1 is differentially expressed in the peritoneum of women with endometriosis, *ID1* expression was quantified by RT-PCR in peritoneal biopsies from women with and without endometriosis. *ID1* expression was increased in the peritoneum of women with endometriosis (*P* < 0.05; Fig. 3B).

### TGF-β1 increases ID1 expression in peritoneal cells and regulates VEGF-A expression through ID1

**Figure 4:**
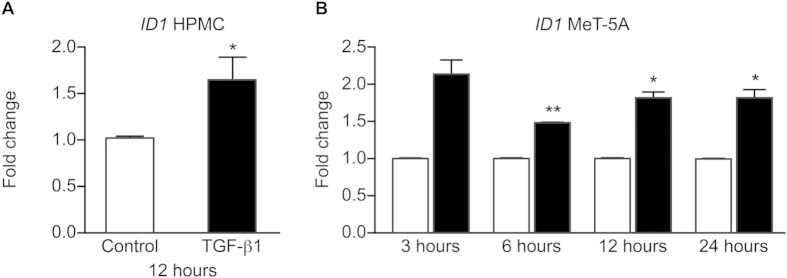
Effect of TGF-β1 treatment on *ID* mRNA expression in HPMC obtained from women with endometriosis and MeT-5A cells. Cells were treated with 2ng/ml TGF-β1 for between 3 and 24 hours. TGF-β1 up-regulated *ID1* mRNA expression in HPMC at 12 hours (**A**) (*p < *0.05 **paired t-test**, n* = *6 each group)*. TGF-β1 also increased *ID1* mRNA expression in the MET-5A at all time points studied (**B**) (*p < 0.05 **unpaired t-test**, **p < *0.01 **unpaired t-test**, n* = *3 each group)*.

We next assessed the effects of TGF-β1 on *ID1 *expression in HPMC and MeT-5A cells. Exposure of HPMC to physiological levels of TGF-β1 for 12 hours increased *ID1* expression (*P* < 0.05; Fig. 4A). Similarly, exposure of MeT-5A cells to TGF-β1 caused a rapid and sustained increase in *ID1* mRNA expression (*P* < 0.05-*P* < 0.01; Fig. 4B).

**Figure 5:**
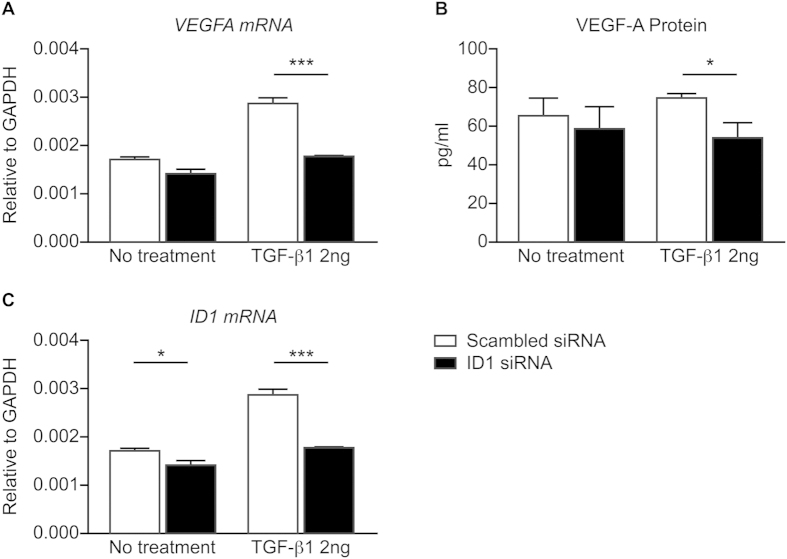
Knockdown of ID1 in MeT-5A cells using siRNA in the presence and absence of TGF-β1. ID1 siRNA significantly reduced *VEGFA* mRNA expression (**A**) and reduced VEGF-A protein secretion (**B**) in TGF-β1 treated MeT-5A cells after ID1 knockdown. TGF-beta1 could not up regulate ID1 (**C**). (*p < 0.05 **unpaired t-test**, ***p < *0.001 **unpaired t-test**, n* = *3 each group)*.

To determine if the molecular regulation of VEGF-A by TGF-β1 is mediated via the ID1 pathway, siRNA was used to knock down ID1 expression in MeT-5A cells. ID1 siRNA significantly decreased TGF-β1-induced *VEGFA *mRNA expression (*P* < 0.001) and VEGF-A secretion (*P* < 0.01) in MeT-5A cells (Fig. 5A,B). Moreover, TGF-beta1 could not up-regulate ID1 after siRNA knockdown of ID1 as compared to the scrambled siRNA treated controls (P < 0.001; Fig. 5C), which supports successful siRNA knockdown of ID1. These data suggest that the regulation of VEGF-A by physiological concentrations of TGF-β1 is ID1-dependent.

## Discussion

Herein, we demonstrate that human peritoneal mesothelial cells are a source of the increased VEGF-A known to be found within the peritoneal fluid of women with endometriosis. We also show that concentrations of VEGF-A positively correlate with levels of TGF-β1 within the peritoneal fluid, and that physiological concentrations of TGF-β1 significantly increase the expression of *VEGFA* mRNA and VEGF-A protein from peritoneal mesothelial cells. In addition, we show that *ID1* mRNA expression is increased in peritoneal biopsies from women with endometriosis compared to women without disease and that *ID1* expression is increased in peritoneal mesothelial cells on exposure to physiological concentrations of TGF-β1. Knockdown of ID1 confirms that it is an intermediary molecule involved in TGF-β1 regulation of *VEGFA* expression and VEGF-A secretion.

Our observation that peritoneal fluid concentrations of VEGF-A are significantly increased in women with endometriosis, compared to women without endometriosis, is in agreement with previous reports^7^,^9^. In this study, we have extended these findings to show that VEGF-A concentrations positively correlate with levels of TGF-β1 in the peritoneal fluid, suggesting that TGF-β1 may regulate VEGF-A expression in the peritoneum. TGF-β is a known regulator of VEGF-A expression during tumorigenesis and we and others have previously shown TGF-β1 to be significantly increased in the peritoneal fluid of women with endometriosis^13^.

We have shown that the peritoneal mesothelium is a source of VEGF-A protein. As the peritoneal mesothelial cells are the largest cell fraction within the peritoneum^20^, it is likely that these cells contribute to the increasing concentrations of VEGF-A within the peritoneal fluid of women with endometriosis described above. Peritoneal mesothelial cells are known to secrete VEGF-A into the extracellular environment in trans differentiation and tumorigenesis where overexpression has been attributed to increased peritoneal fluid concentrations of TGF-β1^21^. Furthermore, macroscopic examination of peritoneal endometriosis lesions, has shown that lesions are highly vascularized and that blood vessels are derived from the surrounding peritoneal tissue, suggesting that expression of VEGF-A in the peritoneum adjacent to endometriosis lesions may play a direct role in neoangiogenesis of endometriosis lesions^22^. HPMC and MeT-5A cells exposed to physiological concentrations of TGF-β1 expressed significantly higher levels of *VEGFA* mRNA transcripts and secreted significantly higher levels of VEGF-A protein, confirming that that peritoneal mesothelium may be a potential a source of increased VEGF-A levels in the peritoneal fluid of women with endometriosis. We believe this may in part explain the induction of neoangiogensis that is observed in the peritoneal tissue surrounding endometriosis lesions^22^.

The IDs are basic helix-loop-helix transcription factors that are transcriptional targets of the TGF-β signaling pathway involved in the regulation of cell differentiation, proliferation and angiogenesis^23^. Overexpression of TGF-β during tumorigenesis has been implicated in the dysregulation of IDs that leads to aberrant cell proliferation, epithelial-mesenchymal transition and neoangiogenesis^24^. In epithelial cells, TGF-β signaling through the Smad 2/3 pathway classically inhibits expression of *ID* genes by activating transcriptional repressor ATF3 which in turns binds to the ATF/CREB site within the *ID* promoter suppressing transcription^25^. However, TGF-β induced over expression of ID1 has been reported in at least one epithelial cell line and in several cancers^26^. Although the mechanisms for this largely remain elusive, one study has shown Smad3 but not Smad2 may be responsible for TGF-β induced ID1 overexpression^27^.

We have previously found *ID1* to be increased in the peritoneum of women with endometriosis using a TGF-β signaling targets gene array^12^. Increased concentrations of TGF-β1 in the peritoneal fluid and peritoneum of women with endometriosis may be responsible for the increased *ID1* expression in the peritoneum of women with endometriosis^12^,^13^. We demonstrated that physiological levels of TGF-β1 significantly increase *ID1* expression in the HPMC and MeT-5A cells. This increase is consistent with a cancerous phenotype as ID1 is reported to be overexpressed in over 20 types of human cancers and ID1 overexpression is associated with poor clinical outcomes in patients with breast, cervical and endometrial carcinomas^24^. As the pathophysiology of endometriosis shares several parallels with tumor onset and progression, TGF-β1 dysregulation of IDs may play an important role in the development of endometriosis lesions. However, further work is needed to confirm that this is the dominant pathway *in-vivo* explaining elevated levels of VEGF-A in women with endometriosis because VEGF-A expression has been shown to be regulated through several different mechanisms in cancer biology^5^,^9^,^10^.

Importantly, we have demonstrated that TGF-β1 increases *VEGFA* expression and VEGF-A secretion through the ID1 pathway in a similar mechanism to that reported in several cancers^28^. IDs are known regulators of VEGF-A expression and a loss of ID function has been shown to lead to a decrease in VEGF-A expression^28^. ID1^+/−^ ID3^−/−^ mice fail to grow tumors due to little or no vascularisation of tumors and blood vessels in these mice fail to undergo neoangiogenesis^19^. As endometriosis lesions result from ectopic tissue implanting and proliferating in a similar fashion to cancer metastasis, the IDs may also play a crucial role in the development of endometriosis lesions.

Endometriosis is associated with chronic inflammation and there is accumulating evidence that key inflammatory factors play an important role in the pathophysiology of this disease^4^. Several of these factors may also play a role in this TGF-β1-ID1-VEGF-A pathway described in this paper. Hypoxia Inducible Factor 1-α (HIF-1α) is a transcription factor known to regulate VEGF expression and several studies have shown ID regulation of VEGF-A to be through HIF-1α^29^. We have previously shown HIF-1α to be increased in endometriosis lesions and the surrounding peritoneum^13^ and therefor it is possible that HIF-1α also plays a key role in TGF-β1 regulated VEGF-A expression. Other inflammatory mediators such as IL-1β, IL-6 and I-CAM1 have been reported to be overexpressed in the peritoneum and are associated with increased VEGF expression and hence neovascularisation in endometriosis^4^. Understanding the role of these and other inflammatory mediators in this pathway may provide a greater understanding of the pathophysiology of this disease.

In conclusion, this study demonstrates a functional role for *ID1* in the peritoneum of women with endometriosis through the overexpression of VEGF-A to potentially increase neoangiogenesis at sites of endometriosis lesions. Blocking the expression of ID1 has been shown to decrease VEGF-A expression and hence angiogenesis during tumorigenesis, and ID inhibitors are being explored as novel therapies for cancers. Thus, ID inhibitors may also be beneficial in the treatment of endometriosis^24^.

## Methods

### Subjects

Ethical approval for this study was obtained from the Lothian Research Ethics Committee (LREC 11/AL/0376). Informed written consent obtained from all patients and all of the methods were carried out in accordance with the approved guidelines. All women included in this study had regular 21–35 day menstrual cycles and none were taking hormonal medication at the time of surgery. All samples used within this study were from the luteal phase of the menstrual cycle which was confirmed by staining the endometrial biopsies with hematoxylin and eosin. Noyes’ criteria was used to determine the cycle phase. In addition, serum levels of progesterone and estradiol further confirmed the cycle phase. All women underwent laparoscopic surgery for the investigation of chronic pelvic pain and peritoneal fluid, primary human peritoneal mesothelial cells (HPMC), peritoneal biopsies, endometrial biopsies were collected at the start of surgery. There were no fundamental differences in the demographics, including; age, BMI, smoking status and presence of other pathologies of the women included within this study.

The women with endometriosis had macroscopic evidence of disease at laparoscopy and this was later confirmed by histology. The women without endometriosis displayed no evidence of endometriosis at laparoscopy and there was no evidence of other underlying pelvic pathology to explain their painful symptoms (e.g. adhesions). Peritoneal fluid (5–10 ml) was collected from women with (n = 8) and without (n = 8) endometriosis and stored in cryovials at −80 °C for later analysis. Primary human peritoneal mesothelial cells (HPMC) were isolated at the time of surgery by gentle brushing the pelvic mesothelium with a Tao^TM^ brush followed by vigorously agitating in 15 ml of serum-containing culture media to dislodge cells, as previously described^30^.

In women with endometriosis, we collected peritoneal biopsies from peritoneum adjacent to endometriosis lesions (2–3 cm from lesion) (n = 3). In women without endometriosis, we collected peritoneal biopsies (0.5 cm diameter) from the Pouch of Douglas (n = 8). After collection, biopsies were divided into two portions with half stored in RNAlater at 4 °C for 24hrs before storage at −80 °C and half fixed in 4% neutral-buffered formalin (NBF) for 24hrs at 4 °C before storing in 70% ethanol prior to embedding in paraffin wax. All peritoneal biopsies collected were studied histologically to confirm the absence of endometriosis. All tissues were collected according to the Endometriosis Phenome and Biobanking Harmonisation Project (EPHect) guidelines^31^.

### Establishment of cell culture

Brushings of HPMC were collected from the pelvic brim in women with and without endometriosis at the beginning of surgery as previously described^29^, by gentle scraping of the pelvic mesothelium (away from the endometriosis lesion in the women with disease) with a Tao^TM^ brush at the pelvic brim (QC Sciences, Virginia, USA). Brushes were vigorously agitated in 15 ml of serum-containing HOSE1 culture media to dislodge cells before transferring to a 75 cm2 culture flask and incubated at 37 °C under 5% CO2 in air (QC Sciences, Virginia, USA). HPMC were cultured as previously described in HOSE1 media containing; 40% media 199, 40% MCDB 105 and supplemented with 15% FBS, 0.5% penicillin/streptomycin and 1% L-glutamine, at 37 °C under 5% CO_2_ in air (Life Technologies Inc., Paisley UK and Sigma Chemical Co., Poole UK)^30^.

The mesothelial cell line, MeT-5A (CRL-9444, ATCC, Middlesex UK), was originally established by transfecting normal human mesothelial cells from the pleural cavity with a plasmid containing Simian virus (SV40) early region DNA, and they express SV40 large T antigen (ECACC, Cambridge, UK). These cells are increasingly used in peritoneal mesothelial cell research and data obtained with MeT-5A cells are thought to be analogous to data obtained with HPMC^20^. The MeT-5A cells were cultured in Iscove’s Modified Dulbecco’s Media (IMDM) ((Life Technologies Inc.) supplemented with 10% FBS and 1% L-glutamine at 37 °C under 5% CO_2_ in air.

### Experimental treatments of HPMC and MeT-5A cells

HPMC cells were plated at 1.5 × 10^5 ^cells/ml, in a 12 well plate with a minimum of five technical replicates per experimental protocol. MeT-5A cells were plated at 2 × 10^5 ^cells/ml, in a 12 well plate, with a minimum of three technical replicates per experimental protocol. Cells were left to adhere for 12 hours before being serum starved for 24 hours. Cells were exposed to physiological levels of recombinant human TGF-β1 (2 ng/ml). As HPMC are known to produce TGF-β ligands, control cell cultures were exposed to a TGF-β neutralising antibody (0.5 μg/ml) for between 3hr and 48hr.

### Experimental treatments of MeT-5A cells with siRNA

MeT-5A cells were plated at 3 × 10^5^ cells/well in a six well culture plate with ID1 siRNA or scrambled siRNA (Table 1) using the neofection transfection method. Two different siRNA sequences were used for optimal knockdown of selected genes of interest (Table 1). Cells were incubated for a total of 48 hours. Physiological concentrations of recombinant human TGF-β1 (2 ng/ml) or TGF-β receptor I small molecule inhibitor (10 μg/ml), SB 431542, were added to cultures 24 hours before the end of the siRNA incubation period. Knockdown of ID1 was performed both in the absence and presence of TGF-β1 (2 ng/ml). Successful transfection conditions were developed using positive control GAPDH siRNA where reduced gene expression was confirmed at the mRNA level by qRT-PCR, at the protein level by Western blotting and cytotoxicity was confirmed to be less than 15% using a lactate dehydrogenase assay (Source Bioscience, Nottingham, UK). Successful ID1 knockdown was confirmed at the mRNA level by qRT-PCR.

### VEGF-A ELISA

VEGF-A ELISA was performed using the Human VEGF-A (DY293B) ELISA Duo set according to manufacturers instructions (R&D systems, Abingdon UK). ELISA plates were read using Lab Systems Multiscan EX Microplate reader at 450 nm with wavelength correction at 540 nm. Samples were quantified using standard curve analysis within the linear range of 16 pg/ml to 2000 pg/ml. Intra-assay CV was 2.5% and the between batch CV was 8.3% for cell culture supernatants and intra-assay CV was 1.9% and the between batch CV is 9.3% for peritoneal fluid.

### TGF-β1 ELISA

TGF-β1 ELISA was performed using the Human TGF-b1 Quantikine kit (DB100B) according to manufacturers instructions (R&D systems, Abingdon UK). Peritoneal fluid and cell culture supernatant samples were assayed for active and total TGF-β1. For complete levels, samples were activated to the immunoreactive form by addition of 1 M HCL for 10 mins before neutralising with 1.2 M NaHO/0.5 M HEPES buffer. All peritoneal fluid complete samples were further diluted 1:2 in calibrator dilutant before addition to the pre-coated ELISA plate, all cell culture and active peritoneal fluid samples were added neat. Standards were prepared and added to ELISA plates before incubated for 2 hours at room temperature with shaking. Plates were washed 4 times in wash buffer and TGF-β1 conjugate antibody added and plates incubated for 2 hours at room temperature with shaking. Plates were washed 4 times in wash buffer before addition of the streptavidin-HRP and incubation for 30 minutes at room temperature with shaking and protection from light. Stop solution was added and ELISA plates were read using Lab Systems Multiscan EX Microplate reader at 450 nm with wavelength correction at 540 nm. Samples were quantified using standard curve analysis within the linear range of 2000 pg/ml to 16 pg/ml. Intra-assay CV is 2.5% and the between batch CV is 8.3% for cell culture supernatants and intra-assay CV is 1.9% and the between batch CV is 9.3% for peritoneal fluid (based upon serum).

### Transcript analysis

RNA was extracted using the RNeasy Mini kit with on-column DNaseI digestion according to the manufacturer’s instructions (Qiagen, West Sussex, UK). First-strand cDNA synthesis was performed using Superscript VILO Master Mix according to the manufacturer’s instructions (Life Technologies). Quantitative (q)RT-PCR reactions were performed on an ABI Prism 7900 Fast system using brilliant III ultra-fast SYBR green QPCR master mix with standard running conditions. Pre-validated primers were used throughout this study and melt curves were analysed to confirm specific products (Primerdesign, Southampton, UK). Messenger RNA transcripts were quantified relative to the appropriate housekeeping gene *GAPDH* as determined by geNorm assay (Primerdesign) and using the 2^−ΔCt^ or the 2^−ΔΔCt^ method.

### Immunostaining

Sections of paraffin embedded tissue were mounted onto microscope slides and dewaxed and rehydrated before antigen retrieval in 10 mM Tris 1 mM EDTA pH 9 with 5 min of pressure-cooking. Slides were washed before incubation with 3% hydrogen peroxide for 30 min followed by blocking in normal horse serum diluted 1:12 in Tris buffered saline with 0.5% Tween 20 (TBST20) for 30 min. Slides were incubated with primary antibody overnight at 4 °C (ID1 Santa Cruz sc-488 diluted 1:1000, VEGF-A Santa Cruz sc-507 diluted 1:100 or isotype match control Rabbit IgG Dako X0903) and then washed in TBST20 before incubation with species specific impress kit for 30 min at room temperature (Vector Laboratories, Peterborough, UK). After washing and incubation with 3, 3’-diaminobenzidine for 5 min slides were counterstained with hematoxylin, dehydrated and visualized by light microscopy, using an Olympus Provis microscope equipped with a Kodak DCS330 camera (Olympus Optical Co., London, UK, and Kodak Ltd., Herts, UK). Due to the limited supply of peritoneal tissue, both positive and negative controls were performed on endometrial tissue.

### Statistical analysis

All results are expressed as mean ± standard error of the mean of a minimum of 3 independent experiments. Quantitative RT-PCR and ELISA were analysed using paired and unpaired students’ *t* tests, as appropriate. All statistical results were generated using GraphPad PRISM version 5 statistical software and a *P* value of <0.05 was considered significant.

## Additional Information

**How to cite this article**: Young, V. J. *et al.* Peritoneal VEGF-A expression is regulated by TGF-β1 through an ID1 pathway in women with endometriosis. *Sci. Rep.* **5**, 16859; doi: 10.1038/srep16859 (2015).

## Figures and Tables

**Table 1:** Table displays siRNA oglinucleotide sequence. All siRNAs were pre-validated and supplied by Life Technologies.

siRNA	Direction	Sequence
ID1	Sense	AGGUGGAGAUUCUCCAGCATT
	Anti-sense	UGCUGGAGAAUCUCCACCUTG
ID1	Sense	CAUGAACGGCUGUUACUCATT
	Anti-sense	UGAGUAACAGCCGUUCAUGTC
